# Multisystem Inflammatory Syndrome in Children

**DOI:** 10.5811/westjem.2022.3.55325

**Published:** 2022-07-11

**Authors:** Muhammad Waseem, Masood A. Shariff, C. Anthoney Lim, Jeranil Nunez, Nisha Narayanan, Kavita Patel, Ee Tein Tay

**Affiliations:** * NYC Health + Hospitals/Lincoln, Department of Emergency Medicine, New York City, New York; † Icahn School of Medicine at Mount Sinai, Mount Sinai Health System, Division of Pediatric Emergency Medicine, Department of Emergency Medicine, New York City, New York; ‡ New York-Presbyterian/Weill Cornell Medical Center, Division of Pediatric Emergency Medicine, Department of Emergency Medicine, New York City, New York; § NYU Langone Health, Department of Emergency Medicine, New York City, New York

## Abstract

Multisystem inflammatory syndrome in children (MIS-C) is an uncommon but emerging syndrome related to SARS-CoV-2 infection. While the presentation of MIS-C is generally delayed after exposure to the virus that causes coronavirus 2019, both MIS-C and Kawasaki disease (KD) share similar clinical features. Multisystem inflammatory syndrome in children poses a diagnostic and therapeutic challenge given the lack of definitive diagnostic tests and a paucity of evidence regarding treatment modalities. We review the clinical presentation, diagnostic evaluations, and management of MIS-C and compare its clinical features to those of KD.

## INTRODUCTION

Severe acute respiratory syndrome coronavirus 2 (SARS-CoV-2) cases emerged in the Wuhan Province of China in December 2019 and were later identified in January 2020 in the United States. Following the spread of coronavirus 2019 (COVID-19) globally, there were reported increases in cases of children presenting with prolonged fever, rash, and conjunctivitis similar to Kawasaki disease (KD).^1^–^3^ Several early case reports noted that a few of these affected children progressed to hypotensive shock, myocardial dysfunction, and multisystem organ failure.^4^–^6^ A report from the Royal College of Paediatrics and Child Health in the United Kingdom (UK) noted that this condition was potentially distinct from KD and categorized this presentation as pediatric multisystem inflammatory syndrome.^7^–^8^ A similar description was established by the US Centers for Disease Control and Prevention (CDC), which termed this condition multisystem inflammatory syndrome in children (MIS-C).^9^ Both organizations have since provided guidance on case definition, evaluation, and management.

Children with MIS-C often present with fever, cough, upper respiratory symptoms, and gastrointestinal symptoms such as vomiting and diarrhea.^10^ We review the epidemiology and clinical presentations of MIS-C, compare and contrast the clinical features of KD and MIS-C, and discuss management options for clinicians who may encounter MIS-C presentations in a clinical setting.

## METHODS

### Literature Review

**Figure 1:**
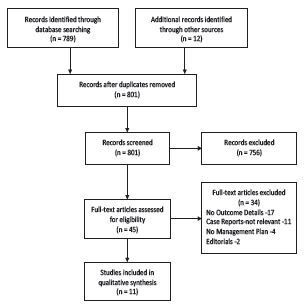
PRISMA flow chart.

In October 2020 we performed a literature search on PubMed along with a web-based search engine using the following Medical Subject Heading terms (performed October 2020): pediatric case reports COVID-19, Kawasaki disease, COVID-19, multisystem inflammatory syndrome in children, pediatric multisystem inflammatory syndrome, and SARS-CoV-2. This search produced 801 records that included scientific articles, editorials, and CDC reports. The sources were reviewed for articles that presented cases with etiology, management plan, treatment, and outcomes as the inclusion criteria for this systemic review. Excluded were records (n = 756) in other languages or without an abstract, review articles, adult-focused articles, pure KD articles, and non-relevant sources. We assessed only full-text articles for eligibility, and 11 studies were chosen for detailed review. The articles were reviewed by pediatric emergency physicians, who selected the articles based on the above inclusion and exclusion criteria. Figure 1 below depicts the PRISMA flow chart obtained from this review.

## RESULTS

### Epidemiology

The first case of MIS-C was reported in the UK in April 2020.^11^ By May 15, 2020, the European Centre for Disease Control and Prevention reported 230 suspected MIS-C cases in 11 European countries. These included the UK, Spain, France, and Italy, along with an additional 12 cases in Canada.^12^ By October 4, 2021, a total of 5217 patients had met the case definition for MIS-C, and 46 deaths related to this condition were reported in the United States.^13^

Early cases of MIS-C were initially described in the UK, the Bergamo region of Italy, Paris, and New York City.^14^–^17^ The median age reported was 7.9 years old, ranging from 1–17 years old.^17^ Most children with severe symptoms of MIS-C were healthy prior to the onset of disease, with asthma and obesity reported as the most common comorbidities.^18^ While initial reports noted a predilection for male gender, subsequent case series with larger sample sizes revealed no significant gender predominance. It is unclear whether the risk of developing MIS-C varied by race, but children and adolescents of African descent represented a disproportionately higher number of cases in the initial UK and French reports.^17^–^18^ In the United States, Black and Hispanic children and adolescents were disproportionately affected by MIS-C, while those of Asian descent comprised a very small minority.^18^

### Etiology

While no direct causal relationship between COVID-19 and MIS-C has been identified, the history of exposure to COVID-19 and the epidemiological spike in cases of MIS-C cases in areas with a high incidence of COVID-19 suggests that the two diseases may be related.^19^ Postulated mechanisms are that SARS-CoV-2 may trigger an exaggerated immune response to the virus among genetically predisposed individuals or may trigger a cytokine-mediated storm.^20^ The suggested hyperactive immune response is characterized by the release of interferons, interleukins, tumor-necrosis factors, and several other mediators as part of innate immune response for clearance of the viral infectious agent.^21^

*Population Health Research Capsule*

What do we already know about this issue?

*Multisystem inflammatory syndrome in children (MIS-C) is a rare and serious emerging manifestation of SARS-CoV-2 as a late immune response diagnosed clinically.*

What was the research question?

*We review MIS-C presentation, evaluation and management, and compare its features to the similar inflammatory syndrome such as Kawasaki Disease (KD).*

What was the major finding of the study?

*MIS-C will require early recognition and initiation of therapy. With a need for monitoring in an intensive care unit, and depending on the severity of the illness and organ system involvement, a multidisciplinary team of specialists need to be on hand during supportive care.*

How does this improve population health?

*This review will aid in its prompt recognition and enable the early initiation of therapy, which has been shown to reduce its morbidity and mortality in all age groups.*

Multisystem inflammatory syndrome in children has been proposed as a post-infectious process occurring during the development of acquired immunity. This concept is supported by the observation that each surge of cases occurring regionally follows the peak of acute COVID-19 infections by 2–4 weeks. The majority of these children had a negative SARS-CoV-2 polymerase chain reaction (PCR) test but a positive immunoglobulin G (IgG) serology, although some had both positive IgG serology and positive PCR tests.^22^ The overt immune response exaggerated by SARS-CoV-2 infection has been theorized to prompt other environmental insults to cause MIS-C in predisposed children and adolescents. A similar mechanism has been proposed as a trigger for KD in the past.^23^

### Clinical Presentation

The most significant clinical features in children with MIS-C are cardiovascular manifestations. Published case studies have reported shock as the initial presentation in at least 50% of cases.^11^,^14^–^18^,^24^–^26^ Patients with MIS-C have been found to present with cardiac abnormalities on echocardiograms such as ventricular dysfunction, coronary dilation, aneurysms, and/or pericardial effusion. Coronary artery changes were reported in 5–48% of patients with MIS-C, while the literature reported lower rates in KD at 20–25%.^27^ Alternatively, about 5% of KD patients presented in shock,^27^ and cardiovascular health in KD patients returned to normal after a few months.

Published case series of MIS-C in the US and Europe have demonstrated common features with KD. Fever is present in all patients with MIS-C with a median duration of 4–5 days.^16^,^25^,^28^–^30^ Patients with KD have fever for five days or longer, although some patients with atypical KD may have fever for a shorter duration. Respiratory symptoms including cough, congestion, sore throat, and dyspnea were reported in 41–66% of cases.^16^,^18^,^25^ While irritability occurred in both MIS-C and KD, other neurologic features of MIS-C included headaches and confusion. 11,14–16,18,25,30.

Gastrointestinal symptoms occurred frequently in MIS-C and less commonly in patients with KD. Most MIS-C case series reported that at least 80% of children with MIS-C presented with abdominal pain, diarrhea, and/or vomiting.^11^,^14^–^16^,^18^,^25^,^26^,^30^–^32^ Coronary artery changes were reported in 5–48% of patients with MIS-C, while the literature reported lower rates in KD at 20–25%. Long-term effects of KD (heart valve dysfunction, arrhythmia, coronary artery aneurysms) were rare, and less than 3% persisted into adulthood.^33^

Skin rashes in MIS-C exhibited variable distribution and presented as maculopapular, scarlatiniform, diffuse erythroderma, or erythema multiforme (Table 1). Patients also had mucosal changes such as cracked, swollen, or erythematous lips, strawberry tongue, or pharyngeal erythema. Additionally, edema of the hands and/or feet were sometimes present. Bilateral and non-exudative conjunctival injection with limbic sparing was also reported. Anterior cervical lymphadenopathy, which is one of the diagnostic features of KD, was also seen in MIS-C but was less common.^14^,^16^,^22^,^30^–^32^

### Evaluation

The diagnostic evaluation of MIS-C herein described is geared toward rapidly identifying life-threatening conditions that require prompt intervention. It is focused on recognizing the constellation of symptoms and findings that are consistent with this syndrome, while concurrently exploring the possibility of alternative diagnoses. In the setting of this emerging new syndrome and the possibility of rapid clinical deterioration, a high index of suspicion should be maintained when evaluating children with fever. This is especially the case when coupled with abdominal symptoms or other features commonly observed in children with KD.^32^ Due to widespread quarantine restrictions and school closures during the COVID-19 pandemic, the incidence of infectious etiologies resulting in prolonged fevers decreased significantly. Heightened awareness of MIS-C has led many practitioners to initiate diagnostic evaluations sooner in the course of febrile illnesses.

The CDC guidelines for identifying suspected MIS-C cases have been described as follows: 1) An individual aged <21 years presenting with fever (greater than or equal to 38°C or a subjective fever lasting more than 24 hours), laboratory evidence of inflammation (elevated C-reactive protein (CRP), erythrocyte sedimentation rate (ESR), fibrinogen, procalcitonin, D-dimer, ferritin, lactic acid, lactate dehydrogenase (LDH), interleukin-6 (IL-6), elevated neutrophils, reduced lymphocytes, low albumin), and evidence of clinically severe illness requiring hospitalization, with multisystem (greater than two), organ involvement (cardiac, renal, respiratory, hematologic, gastrointestinal, dermatologic or neurological); 2) AND no alternative plausible diagnoses; 3) AND positive for current or recent SARS-CoV-2 infection by PCR, serology, or antigen test; or COVID-19 exposure within the four weeks prior to the onset of symptoms.^9^

Common laboratory and diagnostic tests are combined with additional testing directed at identifying a hyperinflammatory state, examining end-organ dysfunction, and exploring infectious etiologies to evaluate children with clinical suspicion of MIS-C. A complete blood count and comprehensive metabolic panel is obtained to evaluate for infectious and inflammatory states, metabolic derangements, renal or hepatic dysfunction, and disease processes associated with hypotension and hypoperfusion. Testing for COVID-19 via SARS-CoV-2 PCR should also be performed. Since MIS-C is believed to potentially be antibody mediated, the presence of serum antibodies to COVID-19 should also be determined to aid in confirming the initial suspicion of viral exposure.

A urinalysis may be used to identify the presence of sterile pyuria associated with KD.^30^ Venous blood gas sampling may be useful in determining acid-base status. White blood cell counts with differential, procalcitonin and lactate level determinations can help identify a systemic bacterial infection and sepsis. Inflammatory markers such as CRP, ESR, ferritin, fibrinogen, D-dimer, and a cytokine panel including IL-1 and IL-6 can be used to distinguish among various types of hyperinflammatory processes. Troponin-I and brain natriuretic peptide (BNP) can be used to assess for cardiac involvement and dysfunction.^7^ In KD, platelet counts rise after day 5 of illness, whereas in MIS-C there is typically a drop in the platelet count.^14^ Laboratory findings that are associated with MIS-C are listed in Table 2, below, as an initial laboratory set followed by secondary and confirmatory tests.

Other components of evaluation include an electrocardiogram (ECG), chest radiographs (CXR), and point-of-care ultrasound (POCUS). In addition to serum evaluation for troponin-I and BNP, an ECG is performed to evaluate for cardiac dysfunction and acute myocarditis, manifested by sinus tachycardia, alterations in the PR interval, as well as findings consistent with myocardial damage including diffuse ST changes. The CXRs are used to evaluate for pulmonary infiltrates consistent with acute bacterial pneumonia, acute respiratory distress syndrome, or acute COVID-19 respiratory disease. The POCUS cardiac evaluation may be useful in evaluating global function, which is diminished in myocarditis associated with MIS-C. In addition, changes in the expiratory and inspiratory diameter of the inferior vena cava are indicative of disseminated or cardiogenic shock.^33^,^34^

### Management

The management of MIS-C in the ED begins with the overall evaluation and classification of the patient into categories based on symptom severity: mild, moderate, or severe. Current treatment of children with severe presentation consists of supportive care with fluid resuscitation and inotropic support, along with directed therapy for respiratory failure, cardiac dysfunction, a hyperinflammatory state, and evaluation for possible thrombosis. In our review, the use of immunoglobulins, corticosteroids, or anticoagulants may be indicated based on the clinical symptoms and the presence of elevated serum inflammatory markers. Application of antiviral and immune modulator therapy remains variable. Early management strategies should be developed in consultation with experts in pediatric infectious disease, intensive care, cardiology, rheumatology, and hematology. Table 3 below includes a summary of early pediatric MIS-C case series with information on clinical presentation, as well as treatment agents used by the authors listed.

### Admission Criteria

Clinicians should maintain a high level of suspicion for MIS-C in children with known or suspected COVID-19 infection who develop fever for several days with no identifiable cause or who have developed shock. Hospitalization may be necessary for patients with suspicion for MIS-C who have significantly elevated serum inflammatory markers, even if symptoms are mild, as some patients may rapidly decompensate and require aggressive resuscitation. Patients who are ill appearing, hemodynamically unstable, require invasive respiratory support, or have evidence of end-organ dysfunction should be admitted to a pediatric critical care unit.

### Shock

Patients with severe MIS-C can develop hemodynamic instability from cardiac dysfunction, as well as a decrease in peripheral vascular resistance. Furthermore, secondary bacterial infection can also lead to sepsis and hypotension. Many children with COVID-19-related MIS-C have presented with shock. In several reports, clinical deterioration occurred rapidly; therefore, the importance of continuous monitoring with frequent blood pressure measurements cannot be overemphasized. Hemodynamic instability should be treated according to established pediatric septic shock guidelines.^35^ Intravenous fluid resuscitation up to 40–60 milliliters per kilogram (mL/kg) in boluses (10–20 ml/kg per bolus) over the first hour with crystalloid fluids should be infused rapidly and titrated to clinical effect. Consider early initiation of inotropic and vasoactive medications for persistent hypotension refractory to fluid resuscitation. Epinephrine and norepinephrine are the preferred initial agents for patients with myocardial dysfunction or decreased systemic vascular resistance, respectively.^35^ Patients with moderate to severe ventricular dysfunction may also benefit from treatment with milrinone and dopamine or dobutamine. Extracorporeal membrane oxygenation (ECMO) may be required in patients with persistent cardiogenic shock refractory to all other treatments. Among the case studies that were reviewed, 11 (9.5%) patients with MIS-C had a presentation of cardiac dysfunction that required ECMO.^16^

Intravenous immune globulin (IVIG) has been used as a first-line treatment in most cases of MIS-C with an overall improvement in cardiac function and decreased inflammatory state. Patients in some case reports with IVIG resistance received a second dose of IVIG, with or without corticosteroids.^15^,^18^,^30^

In children who are ill-appearing with fever, hypoxia, and hemodynamic instability, IV fluids should be initiated immediately, and the child should be admitted to a critical care unit. A CXR is recommended initially to identify interstitial pneumonia and cardiac enlargement. Shock evaluation should include an ECG and echocardiogram to evaluate ventricular function.

Patients who have features of MIS-C without shock or cardiovascular dysfunction (fever, rash, lymphadenopathy, increased CRP/ESR, thrombocytopenia, lymphopenia) should be evaluated with both a laboratory panel and a cardiac evaluation (ECG, echocardiogram). It is recommended that these patients should be admitted to an inpatient unit.

Patients with clinical manifestations and abnormal laboratory findings consistent with MIS-C should be hospitalized for close monitoring and supportive care. Children with severe MIS-C disease and those with hypotension or shock requiring vasopressor support should be admitted to the pediatric intensive care unit. A multidisciplinary team approach involving cardiology, infectious disease, immunology, rheumatology, hematology, and intensive care can be helpful in optimizing patient outcomes in an inpatient setting. Thus far, the long-term impact and sequelae among survivors are not known, and careful long-term follow-up is needed to assess future cardiac function.

### Differential Considerations

Other differential diagnoses to consider are related to bacterial and viral infection. Staphylococcal and streptococcal toxic shock syndrome (TSS) present with fever and shock, and both can present with rash/conjunctivitis, gastrointestinal symptoms (abdominal pain, diarrhea) as a close resemblance to MIS-C, whereas the latter will have cardiac and respiratory features.^36^ While streptococcal infections can demonstrate a strawberry tongue, which can be seen in MIS-C and KD, the lips are usually normal, and the oropharynx demonstrates tonsillar exudate and palatal petechiae.

Common viral infections such as enterovirus, adenovirus, parvovirus, and measles can mimic some features of MIS-C including fever, rash, and conjunctival injection. Gastrointestinal symptoms such as abdominal pain, vomiting, and diarrhea may be present in MIS-C, but can also be commonly associated with adenovirus, enterovirus, rotavirus, and Norwalk virus. Epstein-Barr virus (EBV) can cause multisystem organ failure in the central nervous system, liver, lungs, and heart by inciting a hyperinflammatory state similar to MIS-C.^36^ Cardiac dysfunction and myocarditis leading to heart failure may be caused by parvovirus, adenovirus, human immunodeficiency virus, influenza, echovirus, coxsackieviruses, EBV, and cytomegalovirus.

### Additional Immune Modulators

One of the clinical features and potentially poor prognostic indicators of MIS-C is markedly elevated inflammatory markers and pro-inflammatory cytokines. The efficacy of immune modulatory therapies such as IL inhibitors (IL-1, IL-6), corticosteroids, or convalescent plasma from patients who have already recovered from COVID-19 remains unclear for patients suffering from MIS-C. Anakinra, an IL-1 receptor antagonist, has been used successfully in the treatment of highly refractory KD.^3^,^37^–^39^ Belhadjer et al reported that three children with COVID-19-related MIS-C cardiac dysfunction were successfully treated with anakinra for persistent severe inflammatory state.^18^ According to Waltuch et al one patient was treated with both anakinra and tocilizumab, an IL-6 inhibitor for atypical KD presenting with TSS features.^33^ Four patients had evidence of MIS-C cytokine release syndrome (cytokine storm and significantly elevated levels of IL-6) and they received tocilizumab, an IL-6 inhibitor.^16^,^33^)

Convalescent plasma has been used in a small number of children with SARS-CoV-2 infection with some beneficial results, although its effectiveness is unclear in patients with MIS-C.^40^ Anakinra and tocilizumab may be alternative options in children with severe MIS-C and in patients with markedly elevated pro-inflammatory cytokines, who do not respond to IVIG and corticosteroid treatment.^39^ Management options should be discussed in consultation with pediatric rheumatology and immunology healthcare professionals. Responses to treatments include the normalization of vital signs, the resolution of symptoms, and a decrease in inflammation.

### Anticoagulation Therapy

Acute infection with SARS-CoV-2 has been associated with an increased risk for thrombotic complications due to an amplified inflammatory response and a state of hypercoagulation.^41^ Anticoagulation therapy has been recommended for patients with elevated D-dimer levels or evidence of thrombosis. Currently, there are no definitive guidelines for anticoagulation therapy in children with MIS-C. Patients with either typical or atypical KD should be treated with acetylsalicylic acid. Additional anticoagulation therapy with either a second antiplatelet agent or a systemic anticoagulant (low molecular weight heparin or warfarin) is warranted in children with evidence of medium to large coronary artery aneurysms or existing thrombosis.^3^ Anticoagulation therapy should also be considered for children with MIS-C and myocardial dysfunction, or cytokine release syndrome. A case-by-case management plan should be developed in consultation with pediatric hematology professionals.

### Antiviral Therapy

While some children with MIS-C have evidence of acute SARS-CoV-2 infection at the time of diagnosis, a large number of children have tested negative on PCR and are positive for serum COVID-19 antibodies. Therapy with the antiviral agent remdesivir has been used in ≥12 years patients with severe complications from acute SARS-CoV-2 infection. However, the risks and benefits of this antiviral treatment remain uncertain. Its benefit is not indicated as MIS-C is considered an immune-mediated phenomenon that occurs weeks after a primary SARS-CoV-2 infection. Consultation with pediatric infectious disease and critical care experts on the use of remdesivir is recommended if suspected or confirmed MIS-C patients are not responding to other treatment options.

### Prognosis and Follow-up

Published management guidelines for MIS-C emphasize both short- and long-term follow-up for MIS-C patients who were admitted to the hospital. Close follow-up is recommended for all patients with KD features, cardiac dysfunction, or evidence of coronary artery abnormalities. Repeat echocardiograms should be obtained to screen for coronary artery dilation, aneurysm, and thrombus formation. Patients should be followed up by a primary care physician within a week after discharge from the hospital. Furthermore, follow-up with specialists in infectious disease and rheumatology should also be considered.

## DISCUSSION

Multisystem inflammatory syndrome in children differs from other inflammatory disorders such as KD by its novelty in clinical presentation and patient demographics. The number of COVID-19 cases in children continues to rise, with new variants emerging frequently. With this rise in pediatric COVID-19 cases is an expected rise is MIS-C cases; however, the effect of new SARS-CoV-2 variants on the development and progression of MIS-C is unknown. The need for strict MIS-C identification and management guidelines is thus imminent. Mild to severe cases with cardiac dysfunction require early recognition and management for successful prognostic outcome, and for the majority of patients outcomes are generally good with little to no significant medium- or long-term sequelae.^42^ Vaccinations are now approved for younger individuals; however, the impact of this intervention on the development of MIS-C following another COVID-19 surge is unclear.

## LIMITATIONS

Multisystem inflammatory syndrome in children is a relatively new condition; thus, there is limited literature available to review. As the pandemic evolves and additional waves of infection spread, the identification and management of MIS-C will continue to change. We have attempted to identify the highest quality published evidence and methodology described within this small pool of limited literature. Hence, it has been difficult to draw any definitive conclusions regarding the overall generalizability of our findings.

## CONCLUSION

Multisystem inflammatory syndrome in children should be suspected in children presenting with fever and the symptoms described above following the diagnosis of, or exposure to, SARS-CoV-2. Kawasaki disease has similar clinical characteristics when compared to MIS-C. Early recognition and testing of suspected MIS-C patients will result in earlier treatment initiation and will likely limit the patient’s generalized immune-mediated decline. Managing children with respiratory and cardiovascular dysfunction requires caution and continued surveillance. Long-term studies are required to determine the association of MIS-C with COVID-19 infection and the effect of COVID-19 vaccination on MIS-C, and to understand the effect of new SARS-CoV-2 variants on the development and progression of this condition.

## Figures and Tables

**Table 1:** Clinical characteristics reported in multisystem inflammatory syndrome in children.

Symptoms
Persistent fever
Rash
Vomiting/diarrhea
Abdominal pain
Headache
Cough/shortness of breath
Irritability
Physical Findings
Rash: maculopapular, erythroderma, erythema multiforme-like
Conjunctivitis: non-purulent, bilateral, bulbar with limbic sparing
Lip swelling or cracking
Strawberry tongue
Palm/sole erythema and/or swelling with or without desquamation

*MIS-C*, multisystem inflammatory syndrome in children.

**Table 2:** Laboratory findings in cases of multisystem inflammatory syndrome in children. Range of mean values reported in currently available literature.

Laboratory test	Result value	Reference range	Admission criteria for MIS-C
Initial laboratory set			
Complete blood count			
White blood cell (per mm^3^)	9.7 – 17.4	4 – 13.5	Increased
Hemoglobin (g/L)	84.5 – 110	111–147	Decreased
Neutrophil count (per mm^3^)	10,955 – 16,000	1,800 – 7,800	> 10,900
Lymphocyte count (per mm^3^)	407 – 1,150	1,000 – 4,800	< 1,000
Platelets (per m^3^)	123 – 499	150 – 350	< 150
Comprehensive metabolic panel			
Sodium (mmol/L)	130 – 131	136–146	< 135
Creatinine (umol/L)	63 – 97	62–133	Mild increase
Albumin (g/dL)	2.1 – 3.2	3.5–5.0	< 3.5
AST (IU/L)	57 – 112	10 – 59	Mild increase
ALT (IU/L)	27 – 119	29 – 33	Increased *
C-reactive protein (mg/L)	229 – 301	0 – 5	Increased *
Erythrocyte sedimentation rate (mm/h)	67 – 72	0 – 30	Increased
Secondary laboratory set			
Lactate dehydrogenase (U/L)	363 – 810	45–90	Increased
Procalcitonin (ng/mL)	14 – 46	< 0.15	Increased
Venous blood gas lactate (mmol/L)	2.8 – 3.6	0.5 – 2.0	> 2.0
Ferritin (ng/mL)	610 – 1,857	12 – 200	Increased
Fibrinogen (mg/dL)	313 – 720	200 – 400	Increased
D-dimer (ng/mL)	2,563 – 4,025	< 500	> 1000 *
IL-6 (pg/mL)	170 – 296	< 1.8	Increased
Troponin-I (ng/mL)	0.045 – 282	< 0.35	Increased
Brain naturetic peptide (pg/mL)	788 – 23,093	< 100	> 100

Reference ranges were obtained from Nelson, *Textbook of Pediatrics* (19th edition).

* Indicates lab values that are significant in Kawasaki disease.

*MIS-C*, multisystem inflammatory syndrome in children; *per mm^3^*, per million cubic meters; *g/L*, grams per liter; *per m^3^*, per cubic meter; *mmol/L*, millimoles per liter; *umol/L*, micromoles per liter; *g/dL*, grams per deciliter; *IU/L*, international units per liter; *mm/h*, millimeters per hour; *mg/L*, milligrams per liter; *U/L*, units per liter; *ng/mL*, nanogram per milliliter; *mg/dL*, milligrams per deciliter; *ng/mL*, nanograms per milliliter; *pg/mL*, picograms per milliliter.

**Table 3:** Case series of pediatric multisystem inflammatory syndrome in children (MIS-C). Summary of currently published data of MIS-C patient presentations, treatments, and outcomes. Cardiac involvement includes myocarditis and ventricular dysfunction. Anticoagulation was administered as heparin or enoxaparin.

Case series Pediatric MIS-C							
	Verdoni et al^15^	Grimaud et al^31^	Belhadjer et al^18^	Riphagen et al^11^	Toubiani et al^30^	Waltuch et al^17^	Cheung et al^16^
Country	Bergamo, Italy	Paris, France	France, and Switzerland	London, UK	Paris, France	New York, USA	New York, USA
Number of patients	10	20	35	8	21	4	17
Median age (years)	7.5	10	10	8	7.9	11	8
COVID-19 PCR or Antibody positive	8 (80%)	19 (95%)	31 (88.5%)	2 (25%)	19 (90%)	4 (100%)	17 (100%)
Classic Kawasaki features	5 (50%)	0	0	0	11 (52%)	0	8 (47%)
Atypical Kawasaki features	5 (50%)	20 (100%)	12 (34%)	8 (100%)	10 (48%)	3 (75%)	5 (29%)
Mechanical ventilation	-	8 (40%)	22 (62%)	5 (63%)	11 (52%)	1 (25%)	0
Shock	5 (50%)	20 (100%)	28 (80%)	8 (100%)	12 (57%)	4 (100%)	13 (76%)
Cardiac involvement	6 (60%)	20 (100%)	35 (100%)	7 (88%)	16 (76%)	4 (100%)	11 (65%)
IV immune globulin	10 (100%)	20 (100%)	25 (71%)	8 (100%)	21 (100%)	3 (75%)	13 (76%)
Corticosteroids	8 (80%)	20 (100%)	12 (34%)	5 (63%)	10 (48%)	-	15 (88%)
IV antibiotics	-	-	-	8 (100%)	18 (86%)	4 (100%)	-
Vasoactive agents or inotropes	2 (20%)	19 (95%)	28 (80%)	8 (100%)	15 (71%)	4 (100%)	10 (59%)
Acetylsalicylic acid	2 (20%)	-	-	6 (75%)	21 (100%)	-	4 (24%)
Anticoagulation	-	-	23 (65%)1	-	-	3 (75%)2	10 (59%)
Immune modulator	0	2 (10%)	3 (8%)	-	-	4 (100%)	1 (6%)
Blood cultures	All sterile	All sterile	-	7 (88%) sterile	All sterile	All sterile	-
ECMO	-	0	10 (28%)	1 (13%)	-	-	-
Fatality	0	0	0	1 (13%)	0	0	0
Coronary artery abnormalities							
coronary dilations	0	0	6 (17%)	1 (13%)	5 (24%)	2 (50%)	0
coronary aneurysms	2 (20%)	0	0	0	0	0	1 (6%)
Median PICU LOS, days	-	4 (1–8)	7 (7–10)	5 (4–6)	5 (3–15)	-	6.4 (3–12)
Hospital LOS	-	-	10 (8–14)	-	8 (5–17)	-	7.1 (3–18)

*MIS-C*, multisystem inflammatory syndrome in children; *COVID-19 PCR*, coronavirus disease 2019 polymerase chain reaction; *IV*, intravenous; *ECMO*, extracorporeal membrane oxygenation; *PICU*, pediatric intensive care unit; *LOS*, length of stay.
